# Supplementary material for: From sequence to enzyme mechanism using multi-label machine learning
Source: BMC Bioinformatics. 2014 May 19;15:150. doi: 10.1186/1471-2105-15-150 (PMC4229970; doi:10.1186/1471-2105-15-150)
Supplement: Additional file 2 — Java code of ml2db. Additional file ml2db_code.tar.gz contains the Java source code to run the multi-label machine learning experiments and save the results to database. The code’s Javadoc is included. [file 1471-2105-15-150-S2.zip › additional file 2/ml2db/utils/doc/uk/ac/ed/inf/utils/package-summary.html]

uk.ac.ed.inf.utils


---


|  |  |  |
| --- | --- | --- |
| **Overview** \| **Package** \| Class \| **Use** \| **Tree** \| **Deprecated** \| **Index** \| **Help** | |  |
| **PREV PACKAGE**   **NEXT PACKAGE** | **FRAMES**    **NO FRAMES**     **All Classes** |  |


---

## Package uk.ac.ed.inf.utils

| **Interface Summary** | |
| --- | --- |
| **Initialisable** | A class having a boolean variable telling whether the initialisation has completed correctly |

| **Class Summary** | |
| --- | --- |
| **EntrezUtils** | Utilities for Entrez services. |
| **FileUtils** | Utils to read, write files etc. |
| **Initialised** |  |
| **LoggerCreator** |  |
| **LogUtils** | Utilities to log error messages |
| **NumberUtils** | Class |
| **PathUtils** | Paths to utilities folders and files |
| **PropertiesUtils** | Utilities to manipulate Java properties and properties text files |
| **ReflectionUtils** | Class |
| **RegExpUtils** |  |
| **TimeUtils** | Utilities to manipulate time objects, create timestamps etc. |
| **Utils** | Various utilities to manipulate vectors of numbers, get a timestamp etc. |

---


|  |  |  |
| --- | --- | --- |
| **Overview** \| **Package** \| Class \| **Use** \| **Tree** \| **Deprecated** \| **Index** \| **Help** | |  |
| **PREV PACKAGE**   **NEXT PACKAGE** | **FRAMES**    **NO FRAMES**     **All Classes** |  |


---
